# Treatise on Softening of the Brain

**Published:** 1843-07

**Authors:** 


### BRITISH AND FOREIGN
### MEDICAL REVIEW,

FOR JULY, 1843.

### PART FIRST.

### Analytical and Critical Reviews.

### Art. I.

*Traité du Ramollissement du Cerveau.* Par Max. Durand-Fardel, M.D. &c. &c.—*Paris*, 1843. 8vo, pp. 526.
*Treatise on Softening of the Brain.* By Max. Durand-Fardel, M.D. &c. &c.—*Paris*, 1843.

The work of M. Durand-Fardel was composed to compete for a prize offered by the Royal Academy of Medicine for the best memoir on the following subject: "Describe the different species of softening of the nervous centres, (brain, cerebellum, and spinal marrow,) their causes, their signs, and their treatment." The volume before us, the result, as its author states, of several years of clinical observation, gained the prize.

Two very distinct things may be understood, observes the author, by the expression *softening* of the brain: either a particular morbid state, a disease to which a new name must be given when its nature shall have been satisfactorily established; or the mere condition of diminished consistence of the cerebral substance. It is of the former that M. Durand-Fardel treats,—in short, of the disease described originally by Rostan.

The disease may be acute or chronic; a division which M. Fardel, and with some justice, as the sequel will show, blames authors for having failed to recognize in respect of its morbid anatomy, although not a few of them have distinguished its acute and chronic forms symptomatically. The acute disease is first considered.

Acute softening. Softening of the brain during its acute stage is specially characterized by redness and diminished consistence, without disorganization, of the affected part. The cases observed by M. Fardel give further demonstration of a fact long known, that acute softening is by far the most frequent in the convolutions. Of thirty-three cases, thirty-one were examples of this seat of the disease, and in nine of them the convolutions were the sole parts affected. Fifty-three cases, collected from various sources, give the following results as regards the situation of the morbid change:

| | |
|---|---|
| Convolutions and white substance | 22 |
| Convolutions alone | 6 |
| White substance alone | 5 |
| Corpus striatum and optic thalamus | 6 |
| Corpus striatum alone | 11 |
| Optic thalamus alone | 4 |
| Pons varolii | 3 |
| Crus cerebri | 1 |
| Corpus callosum | 1 |
| Walls of the ventricles, septum | 1 |
| Fornix | 1 |
| Cerebellum | 1 |

From these figures, and from some further facts referred to by the author, he concludes that both cerebral substances are almost always simultaneously softened; whereas, à priori views might lead, and did actually lead, some writers to teach that the cortical substance suffered most frequently. But M. Andral long since exposed the error; and M. Fardel's merit is simply that of recounting the figures.

The extent of brain implicated *may* be very considerable. M. Fardel has once seen almost the entire surface of both hemispheres and that of the ventricles softened. Such extensive disease is singularly uncommon however, and still more rare is diminished consistence of the entire mass of one hemisphere. We have the opposite extreme in cases where a space not larger than a pea is solely affected. The symptoms are far from being proportional, either in severity or otherwise, to this great difference in extent of anatomical change.

M. Fardel recommends, as the grand means of establishing the degree of softening in the affected tissue, that a stream of water be allowed to fall upon the part from varying heights. We should have expected that "several years of observation" might have led to the discovery of some more accurate standard than any furnished by this rude and, as experience has long proved, unsatisfactory sort of experiment. M. Fardel thinks it impossible to fix with precision the healthy amount of consistence of the brain, so much is this liable to vary with individual organization, the nature of the fatal disease, the composition of the blood, the state of the atmosphere, &c. He considers it easier and not less important to ascertain the relations of different parts to each other in regard of this property, and gives us the following as the results of the examination of 150 brains: The convolutions of the inferior surface of the anterior and middle lobes are much less firm than those of the convexity; this is especially true of the part answering to the sphenoid fossa; here the softness is often so great as to appear at first the result of disease, the pia mater carrying away with it during its removal shreds of the more superficial substance. On the convex surface the convolutions of the middle part are somewhat more dense than those of the anterior lobes, and those of the occipital end of the brain distinctly harder than the others, sometimes to quite an extraordinary degree. These observations, which the author adds to the more common knowledge respecting the relative consistence of the cerebellum, medulla oblongata, pons, and cerebrum, are of importance, and will, we trust, be confirmed; we wish this for the sake of those frequently engaged in examining brains, for we have found such persons, (without, we confess, being ourselves able to remove the difficulty,) very sadly puzzled by this same matter of consistence in not a few cases.

Two important modifications of colour attend the progress of acute softening of the brain: redness first, and a yellow tint afterwards.

*Redness* occurs under three forms; those of injection, of sanguineous infiltration, and of uniform redness. Injection occurs especially in the medullary substance, and in the central parts of the brain, and affects not only the softened part, but those immediately adjacent—the latter, indeed, are often the most abundantly injected. In the greater number of instances, injection, when considerable, is accompanied with infiltration of blood, sometimes with small effusions of this fluid; in other cases the diseased spots have the appearance of patches, irregular in shape and size, red, violet, or blackish in colour. Besides this infiltration, observes the author, there is frequently to be seen a *uniform* red discoloration of the softened parts; this exists in the gray substance in lieu of injection. The tint varies from pale pink to red, never of a deep kind, and appears the result of imbibition rather than of infiltration of blood. There appears to be looseness both of expression and of idea in this mode of stating the varieties of colour of the softened substance; colour is confounded with vascularity and effusion of blood, the effect with the cause. Yet M. Fardel is so enamoured of his arrangement, that he criticises M. Lallemand for establishing the distinction just referred to, which common sense clearly requires. Whatever be its cause, red discoloration is generally much more marked in the gray than the white substance.

The *yellow colour*, often distinguishing softened cerebral substance, is next examined by the author, in respect of its nature and the various aspects under which it presents itself. Here we are favoured with a prolix refutation of M. Lallemand's erroneous notion respecting the necessary dependence of this colour on infiltration with pus: a notion over and over again refuted by experience, and which we scarcely expected to find again made the subject of such detailed exposure. The author himself believes the yellow colour the result of altered hue in blood infiltrated into the softened substance, assimilating it to the tint observed in ecchymoses externally: this is all very well; every one knows that such is the true explanation; but it is really amusing to observe the *naïveté* with which M. Fardel assumes the tone of an instructor in this very simple matter. He states that in cases of violent cerebral congestion he has sometimes seen a slight yellowish tint in the white substance of the hemispheres, "the result of transudation of blood still contained in the vessels," a species of *transudation* which we cannot profess ourselves able to conceive. Among the phenomena of slow poisoning by lead, is a peculiar dirty-yellow colour of the brain, ascribed by M. Tanquerel des Planches to the actual presence of lead,—by M. Martin-Solon connected with the previous existence of delirium, and an hypertrophous state of the brain. This colour M. Fardel suggests may have been the result of repeated congestions.

The period of the disease at which the yellow tint becomes apparent is worthy of investigation. M. Fardel states that it is rarely visible quite at the outset in the cortical substance, on account of the brightness of the red hue. In the medullary substance it is seen earlier either between in jected points, or around partial infiltrations. But, adds the writer, in acute softening the yellow colour is "the result of actual imbibition of blood infiltrated or still contained in its vessels; accordingly it is almost always coupled with redness." We are again at sea; the imbibition by the brain of blood still contained within its vessels is beyond us; be that as it may, however, the important point is that complete yellowness of colour is extremely rare. Andral's eleventh case (Clin. Méd. t. v.) exemplifies this condition in a very satisfactory manner nevertheless.

*Gray discoloration* M. Fardel has never observed in cases of acute softening: neither have we, nor do we expect to find it. A greenish hue he regards, like the rest of the world, as evidence of suppuration or putrefaction. He once observed a remarkable deadness of the white colour of the medullary substance; this condition is the common one in the chronic disease.

*Modifications in the form* of softened parts next engage M. Fardel's attention. Tumefaction is a necessary result of the superabundance of fluids in their interior; this causes dryness of the corresponding meninges, but we have never noticed, nor can we easily understand the production of a particularly tense state of the adjoining dura mater, unless where the tumefaction is very considerable indeed. This state of tumefaction of the softened convolutions has been admirably figured, as many of our readers are aware, by Dr. Carswell. There is nothing new in the author's description, but he connects with this subject an enumeration of the characters of senile atrophy.

Softening of the brain, although sometimes surrounded by injection or infiltration, is generally an accurately circumscribed change; the rest of the organ exhibiting its natural colour and consistence. The meninges, however, appear under very various aspects. The dryness of the arachnoid produced by tumefaction mediately, immediately by the stretching to which the membrane is subjected, has already been mentioned; the surface is sometimes slightly viscid. The conditions of injection of the pia mater observed in twenty-four cases were as follow:

| | |
|---|---|
| No injection or ordinary injection in . . . . . | 13 cases. |
| Tolerably marked injection . . . . . . | 5 ... |
| Considerable ditto . . . . . . . | 4 ... |
| Injection limited to the diseased hemisphere . . . | 1 ... |
| Injection limited to the point of the surface corresponding to the softened part . . . . . . . | 1 ... |
| General suffusion of pia mater . . . . . | 2 ... |

In thirteen cases of M. Andral's publication, there were only five in which the pia mater attracted attention by undue vascularity; so that among thirty-seven cases, six only furnished examples of very notable injection. The pia mater would appear from these facts to exercise very little influence in the production of softening.

M. Fardel's observations (as, indeed, we should have stated before) were made upon subjects of advanced age, in whom, as is well known, atrophy of the convolutions, (a far from uncommon state, and admirably described by Dr. Carswell,) is accompanied with *accumulation of serosity* in the anfractuosities. When M. Fardel found this state of atrophy coexistent with serous effusion in the pia mater in cases of softened brain, he correctly regarded the effusion as unconnected with the softening.

When, on the contrary, the convolutions are of natural size and development, he considers it certain that, if serosity be present, it depends upon a morbid action, and is not produced simply to fill the space which would otherwise be empty from the shrivelling of the convolutions. M. Fardel regrets that he cannot in the present place disclose his reasons for adopting this view of the relations of the effused fluid and diminished volume of the convolutions in senile atrophy: we regret it also, for this is precisely the most interesting and debateable point at which the author has yet arrived in the course of his descriptions. No allusion is made to Dr. Carswell's opinions on this subject.

The more closely the liquid approaches simple serosity in its characters, the less important is it pathologically. The gelatinous-looking appearance it sometimes presents depends simply on its being infiltrated in the meshes of the cellular membrane lying between the arachnoid and pia mater. M. Fardel did not meet with a single example of accumulation of serosity in the pia mater having such characters as to prove its origin recent, nor in any case did he discover pus; the cases published by M. Rostan give a similar result; and in a single one only of the thirty-three published by M. Andral was a small quantity of slightly turbid serosity found under the arachnoid. These facts seem to demonstrate a much greater amount of independence on the part of acute softening and affection of the meninges than we should, à priori, have been led to expect. M. Fardel differs altogether from those authors who regard the thickening and opacity of the arachnoid, frequently observed in aged subjects, as capable of explaining the symptoms attending softening during life.

The *adhesions* found between the pia mater and brain are of three kinds, according to this author; they are produced by: 1, a sort of viscosity poured out between them, when no actual liquid is interposed between them, as in cases of compression of the brain and flattening of the convolutions; 2, the vessels passing from the membranes into the brain; 3, old or recent cellular adhesions. M. Fardel appears to have paid attention to a point concerning which much difficulty is frequently experienced at post-mortem examinations, the natural amount of adhesion of the membranes to the subjacent brain at the different points of its surface; we therefore condense the results at which he has arrived. He thinks it may be established as a general proposition, that when the examination is made within forty hours after death, and the temperature not very high (under 77° Fah.), erosion of the surface of the brain, produced in removing the pia mater, is a morbid phenomenon. Cases in which putrefaction has advanced with very notable rapidity are of course to be excepted; and the author would except from the mass of the brain, under all circumstances, the convolutions of the middle and sometimes of the anterior lobe, the natural softness of which renders the separation of the meninges extremely difficult. The meninges are generally easily removable over the convex and lateral surface of the hemispheres; however, some difficulty is met with in detaching them at the union of the upper and internal surfaces of each hemisphere. The firm and narrow convolutions forming the posterior points of the hemispheres are so closely united, that serosity is never found infiltrated between them; the removal of the membranes is consequently very difficult here, for, generally speaking, the facility of removal is directly as the quantity of serosity in the pia mater.

But, as must have struck every one who has raised a patch of meninges and found particles of brain clinging to its under surface, this adhesion may depend either upon the cohesion of the cerebral substance being so much diminished, that it yields under the traction of a naturally adherent pia mater, or upon increased firmness of adhesion of this membrane. Here is a difficulty of which we have often felt the importance. M. Fardel does little to remove it. In general, in cases of acute softening, unless when the disease was very recent, he fancies he found true adhesions, (not of plastic lymph except in the rarest instances,) " consisting in a rather close agglutination of the softened pulp to the pia mater." This is anything but clearly intelligible.

Seventeen cases of acute softening, the great majority of them reported by the author, next follow; and upon the information furnished by these he proceeds to inquire into and minutely consider the nature of the essential elements of the anatomical changes we have already passed in review; congestion, sanguineous infiltration and inflammation.

*Congestion of the brain* is anatomically characterized in the gray substance by uniform pinkish discoloration, (the author ought to have distinctly said *inflammatory* congestion ;) in the white substance by punctiform red injection, and sometimes by patches of reddish staining. M. Fardel considers that it is easy to distinguish active congestion from inflammation in the brain as elsewhere, by the circumstance that in the former state the natural consistence of the part is either natural or slightly increased. MM. Bouillaud and Gendrin, (both fanciful persons, more especially the deputy for Angoulême,) appear to have been the sole observers of an *increase* of the natural consistence under these circumstances. Tumefaction indisputably occurs in simple congestion.

"*Sanguineous infiltration*" is introduced to our notice with a very remarkable flourish of trumpets, so loud and prolonged that we fancied in our innocence the excellent author had discovered a key to the whole pathology of the brain. But the thing is not altogether new, seeing that, according to the author himself, it has been described by Cruveilhier as capillary apoplexy, by Rostan, by Lallemand, MM. Fantonelli, Bravais, Didey, and many other distinguished Frenchmen, whom no one ever heard of before, and seeing further that his first case is taken from Dr. Abercrombie's book. But all these men, with the exception of MM. Fantonelli, &c., were wrong, according to our learned friend, in not studying the anatomical state in question as a distinct and separate condition capable of existing independently of all others. In 1840 appeared M. Fardel's own thesis, which contained " all that medical science possessed on the subject," (that is, according to the impartial estimate of its author,) and numerous original observations.

" Sanguineous infiltration" occurs in very different forms. Sometimes small collections or spots, " smaller *or* larger than a millet-seed," (why we could have guessed *this* ourselves,) scattered here and there through the brain or grouped together, occurring most commonly in flat patches on the surface of the convolutions, characterize this morbid state. In other cases rounded masses, about as large as a nut, single or in numbers, are observed. In a third form, " which may be called diffuse infiltration, the latter has no determinate appearance, and presents the greatest variety of aspects and sizes." All these forms are characterized by the presence of blood in the cerebral substance, and mixed, more or less, intimately with this. Afterwards, we are told, the blood is " combined with the cerebral substance," (p. 61 ;) as if this condition and a mixture of the blood with broken-up brain were one and the same thing. Be this as it may, M. Fardel regards sanguineous infiltration of the three kinds described, "as the result of congestion accompanied with the rupture of vessels ;" we cannot for the soul of us see (viewing the matter in the light he does, and speaking of broken-up brain,) how he could regard it as anything else. The only point which it would have required the least perseverance or acumen to determine, namely, whether any, and if so what, particular state of the vessels causes rupture to occur in some cases as a consequence of congestion, while in others it passes off without any such result, the author avoids examining, because he might be led thereby into a discussion rather foreign than otherwise to his subject. We apprehend, if he had known much about the matter, he would scarcely have been deterred from an exhibition of his knowledge by any qualms respecting prolixity or want of connexion with his main subject. But the reader will observe that the occurrence of simple transudation through the vessels is not considered.

The condition of the brain affected with this species of hemorrhage, for it is nothing more, however pertinaciously M. Fardel may strive to decorate it with a new title,—varies in respect of firmness. The affected part may be of natural consistence, harder or softer than in health. The induration produced in this way is worthy of consideration, because M. Cruveilhier, with a want of judgment scarcely worthy of his reputation, ascribes it to inflammation. It is obviously produced in precisely the same way as the induration in pulmonary apoplexy; the pouring forth of a quantity of liquor sanguinis into a circumscribed space, amidst the intervascular spaces of a tissue, could scarcely produce any other *primitive* result, provided only the quantity extravasated were sufficiently abundant.

Blood being once infiltrated into the cerebral substance, inflammation generally follows around it; an inflammation produced either because the primary congestion, inflammatory in its nature from the first, follows its natural course to inflammation, or because the effused blood acts as an irritating agent on the tissue into which it has passed. Once softening is developed, " sanguineous infiltration" becomes a state of secondary importance in general, and its history but a part of that of encephalitis and of softening.

A morbid state which always commences by congestion or sanguineous infiltration, is essentially characterized by softening and often attended with tumefaction, establishment of adhesions, &c., must be considered inflammatory in its essence ; *red* softening is, in other words, an evidence of encephalitis. And not only of encephalitis, but, according to M. Fardel, of this affection in its *acute* stages ; *colourless* softening being the same malady in a *chronic* state. If the first part of this proposition be old, and the doctrine commonly professed, not so the last ; this is indeed the most important point started by M. Fardel, and, as we advance, we shall see the amount of evidence adduced to substantiate a view, certainly in its details at least, both novel and important.

It is difficult to assign very accurate limits to the acute stage; the author nevertheless is enabled to conclude from the analysis of a number of cases that redness very rarely disappears before the twentieth, or remains apparent after the thirtieth day. Acute softening may in some rare cases exhibit itself without redness; in some of these the colour is yellow, as before described, in others suppuration exists.

In many cases of softening of old standing, no trace of vascularity can be discovered; in the majority such traces are very slight; in some, however, numerous well-developed vessels exhibit themselves. These statements are justified by the examination of upwards of 200 cases, the product of the observation of Rostan, Andral, Lallemand, Raikem, and the author. Among this large number of cases there were only four evident examples of chronic softening accompanied with redness; and these only of acute softening unattended either with redness, yellow discoloration, or suppuration. To the question of the anatomical characters of "chronic softening," we shall return by and bye.

*Symptomatology of the acute disease.* All cases of acute softening may in respect of their symptoms be referred to one of two groups: the one characterized by weakness or abolition of the cerebral functions, the other by perversion or excitement of these functions; in the one case the disease follows the course of hemorrhage, in the other of meningitis. Further comes a number of cases "presenting an association of the characters of both groups, and capable of being placed indifferently in one or the other of them."

Beginning with the beginning, M. Fardel inquires into the correctness of the statements of Professor Rostan respecting the *premonitory symptoms* of the disease, symptoms made by that writer to constitute the first of its "two periods." These premonitory symptoms, according to Rostan, are cephalalgia, vertigo, weakness of the intelligence, changes of temper, tendency to sleep, numbness, formication, sometimes stiffness and pain in the limbs; on other occasions delirium and general agitation; lastly, still according to the same writer, mental alienation and senile dementia often precede softening, to which must be added impaired vision amounting even to perfect blindness, tinnitus aurium, &c. Among these symptoms, put forward as premonitory, appear some, we perfectly agree with M. Fardel, which are evidence of the actual existence of the disease itself, of the existence of softening; for example, delirium, stiffness of the limbs, loss of vision, &c. Others among them, as cephalalgia, vertigo, tendency to sleep, numbness, &c., do actually occur before the development of the disease, and are premonitory; but they may be absent altogether, and cannot under any circumstances be regarded as signifying a *period* of the disease. It is evident, too, that these premonitory symptoms, depending as they do upon cerebral congestion, a state which may precede all varieties of affections of the brain, cannot where they exist have any direct importance as signs of coming softening. Nor are they important from the frequency or regularity of their occurrence. The cases in M. Rostan's book furnish a greater number of examples of absence than of presence of such symptoms, and M. Fardel's own experience, as condensed in the page before us, justifies the assertion that, as is the case with almost all acute maladies, premonitory symptoms may occur in softening, but do not constitute, in the instance of either a special and characteristic period of the disease. In numbers of M. Fardel's cases the affection set in perfectly suddenly; and if it be admitted that in some of these instances premonitory symptoms may really have existed, but not been noticed by the friends or others who were alone able to give any account of the patients, (and this *was* probably the fact, as M. Fardel admits,) it must surely in turn be conceded that symptoms attracting so little attention cannot *practically* be esteemed of great importance. Andral distinctly admits that premonitory symptoms may be absent in the phrase, "softening *may* exhibit a premonitory stage." (Clin. Méd. t. v. p. 582.) We are anxious to impress these facts upon the minds of our readers, because we have met with many persons who conceive that softening is to be readily distinguished by the character of the attack as respects suddenness or gradual development; where a great number of cases of softening and of hemorrhage are compared in respect of this point, no very distinctive character, concludes M. Fardel, is observed in one or the other category. Long since we instituted, for our own information, a similar comparison, and came to a precisely similar result. The author adds, however, that numbness, cramp, formication, weakness, when limited to one side of the body, or especially to one limb, are of all signs the one most valuable as premonitory of softening; a statement, nevertheless, very materially qualified by the addition that when observed they commonly belong to softening actually established. A final corollary from his facts is given by M. Fardel as follows: when acute symptoms declare themselves in an individual already labouring under chronic softening, it is very probable that they depend on superadded acute softening, because the occurrence of hemorrhage under these circumstances is extremely rare.

*Invasion.* In some cases the disease sets in by general or partial weakening of the intellectual faculties, of movement, and of sensibility. These phenomena lead gradually or by sudden advances to total annihilation of these faculties and properties. In other cases the symptoms of softening set in instantaneously and are constituted by the simultaneous appearance of the same series of phenomena; here is the *apoplectiform* variety. In certain instances, the softening announces itself from the commencement by phenomena of excitation, by spasmodic symptoms connected or not with those of paralysis or collapse: and these phenomena may affect the intelligence only, or the functions of movement alone, and consist, for example, of epileptiform attacks, but such symptoms are far from occurring in the greater number of cases. In 137 cases, collected from his own portfolio and from several other sources, the invasion of the disease was apoplectiform 79 times; 58 times it was of a different kind.

But the apoplectiform variety being considerably more common in the cases of Rostan and of the author, who observed only subjects of very advanced age, than in those of Andral and of others whose experience bore upon subjects of all ages, from ætat is fifteen upwards, it is natural to inquire whether the frequency of that form in the cases of the former writers may not depend upon the age of their patients. This question must be answered in the affirmative, as some other considerations, which it is needless to explain, satisfactorily show.

*Disorders of movement* next engage the writer's attention. In almost all cases of softening paralysis occurs, commonly limited to one side of the body, sometimes to a single limb. Complete or incomplete, it is occasionally accompanied with persistent contraction (contracture),—a contraction which may amount to no more than slight stiffness, or be so powerful as to be with difficulty overcome by the observer. Stiffness of the joints may occur on the nonparalysed side; under these circumstances, or when the paralysis is very imperfect, there is a fear of mistaking a voluntary or simply automatic contraction, if the intelligence be deeply affected, for a morbid stiffness. Instead of paralysis convulsions may be observed, sometimes general and simulating those of epilepsy, sometimes partial; in some instances tetanic contraction, or simple muscular trembling, &c. These varieties of morbid mobility may alternate with paralysis, precede, follow, or exist without it: sometimes they are only noticed on the side opposite the paralysis. The relative frequency of these different conditions is shown in the subjoined enumeration:

In 32 cases of acute softening:

| | | |
|---|---|---|
| Paralysis occurred in . . . | 23 cases. | |
| general in . . | 2 | =23. |
| Simply weakened power of motion in . | 1 | |
| Paralysis limited to the arms in . . | 6 | |
| affecting one entire side in . | 14 | |

M. Fardel, in accordance with common experience, never saw the lower more completely paralysed than the upper limb; whereas the converse he frequently observed. A certain harmony is noticed between loss of movement and loss of intelligence; when the disease sets in by a sudden loss of sense, the hemiplegia is in general sudden and complete, or nearly so. In seven cases only did M. Fardel discover stiffness of the paralysed limbs; in five of these it appeared with the invasion, and had increased in two, diminished in one, and disappeared wholly in two cases by the following day. Numerous other varieties in these respects were observed by the author, but we must refer for them to his own pages.

Several of those who have written on acute softening furnish no examples of integrity of the faculty of movement: on the other hand, Raikem, Andral, Lalesque, Faber, and the author, relate cases in which the intellectual faculties alone suffered.

In every one of upwards of two hundred cases of paralysis depending upon various causes, the author invariably found the loss of motion seated on the side opposite that of the cerebral disease. And though there are, indubitably, cases on record in which both were on the same side, these cases are sufficiently rare to render it perfectly incorrect to speak of their occurrence as in any measure frequent,—which appears, nevertheless, to have been rather recently done by M. Rostan. (Gazette des Hôpitaux, 12 Juin, 1841.)

The section on the *state of sensation* in acute softening is introduced by some observations upon the difficulty of forming an accurate judgment respecting the condition of that property. Signs of sensation necessarily exhibit themselves and disappear in the same spot at different periods of the day; besides, there is no small difficulty in distinguishing, in certain cases, simply automatic movements from those provoked by pain, or the cries resulting from real suffering from the inarticulate sounds uttered by patients under the influence of an apoplectiform attack.

Sensation, when developed in a paralysed limb, may give evidence of its existence by moans, by grimaces, by the approximation of the healthy arm to the paralysed side, or by movements of the paralysed limb. But M. Fardel is sufficiently acquainted with the writings of Dr. Marshall Hall to know that movement may occur in limbs perfectly paralysed in respect of voluntary motion and sensation. The precise words of the writer may be worth extraction: "It is certain that in a good number of cases in which sensation and motion were so completely abolished in the limbs that a pin might be driven with impunity in its whole length into the flesh, I have seen movements take place, when the same experiment was repeated in the palm of the hand, the sole of the foot, the internal surface of the forearm, or around the ankles; but almost invariably, besides the slight movement of retraction of the limb, the patients gave some sign of sensation, as moans, or more especially a movement of the opposite arm towards the point irritated. It is important to make these remarks, because they suppose [i. e. the facts they refer to,] a connexion between sensation and volition, and, on the other hand, the production of these movements. It has occurred to me several times, however, as was noted in the experiments of Dr. Marshall Hall, not to be able to detect any sign justifying even a suspicion of consciousness of motion on the part of the patients." The manner in which all this is stated shows how imperfectly Dr. Hall's Memoirs are appreciated by the profession generally in Paris, and indeed M. Fardel very patronisingly admits, that "*perhaps* they are not sufficiently known" in the centre of the civilized world, as his countrymen very modestly term their capital town.

Loss of sensation (anæsthesia) commonly coexists in acute softening with that of movement; but is somewhat less frequent than the latter. This proposition is the expression of general experience,—but M. Fardel's own cases, which give nine instances of retained sensation among twenty-three of paralysis, furnish a higher proportion of the former than we believe to be usual.

*Exaggerated sensibility* of the skin, or deep-seated pains in the limbs, rarely occur in *acute* softening; they are more common, as we shall see, in the chronic disease. In the former affection they are so rare that the author, out of upwards of two hundred cases, has only been able to find some five or six instances in which they occurred. But there are certain modifications of sensibility more frequently observed in acute softening,—these are numbness, formication, and disagreeable or even painful pricking sensations, seated either in the limbs or face, rarely in the trunk. These may occur either as premonitory symptoms, at the commencement or during the course of the disease, when the paralysis is incomplete, or when it has begun to diminish in intensity.

*A sensation of deep-seated cold* in the paralysed limb is "very common," according to this writer, at the commencement of the disease; sometimes this occurs before any other direct symptom, and if accompanied with even vague and indecisive signs of cerebral congestion, is far from being without diagnostic value.

*Cephalalgia*, says M. Lallemand, is one of the most constant premonitory symptoms of encephalitis; it continues during the first period of the disease, &c. M. Fardel has very meritoriously exposed the error of this statement, if received without limitation: in sixty-seven cases collected by him from different sources, cephalalgia was noted only eighteen times, and, in some of these instances, appeared to depend either on the preexistence of chronic softening, or upon some accessory circumstance. M. Lallemand's patients were generally younger, however, than the others, and the encephalitis, in their cases, attended with meningitis. In some of M. Lallemand's cases there existed disease of the cranial bones, or the softening was traumatic,—all circumstances manifestly modifying the pathological character of the malady. The general result would then appear to be, that cephalalgia is not a symptom of the great importance which it is usually believed to be; "it is frequently absent, and when it does exist presents no peculiarity either in respect of seat or nature." Yet assuredly the next sentence of the author very seriously qualifies the signification of this latter clause: "It is nevertheless certain, that persistent cephalalgia of some intensity, especially if confined to a given spot in the head, may become a valuable means of detecting either a threatened or actually existing softening."

*The aberrations of intellect* observed in this disease are very various. In some cases, several days before the invasion of softening, the character or the intellectual faculties present some slight modifications; irascibility or sadness, or, on the other hand, dulness and confusion of ideas, have at that period been noted. When the disease is gradually developed, a gradual weakening of the faculties, which may advance to a perfect state of hebetude, or actual coma occurs,—or agitation, loquacity, delirium. Coma, in other instances, is established from the first, at least such is M. Fardel's experience, in opposition to the statement of Rostan, that it scarcely appears until the second stage.

The disease may set in by sudden hemiplegia, without any change in the intellectual faculties; this is rare, but less so in softening than in hemorrhage,—a point of diagnosis to which we shall return.

The frequency with which softening declares itself by loss of sense, and coma, from the first, has led many authors to speak of the disease under the head of apoplexy,—one illustration among a thousand of the grave errors committed by *soi-disant* practical men, who content themselves with the merest outward similarity as evidence of the identity of diseases. "The treatment is the same," say these wiseacres; "what matters aught beyond this?"

In all cerebral affections the *condition of the face* is of extreme importance, both in respect of its expression and of the position of its different parts. In speaking of the deviation of the commissure of the lips, M. Fardel notices a source of error in diagnosis worth being borne in mind: "When the teeth fall out the mouth habitually loses all symmetry, and is drawn or rather falls towards the side upon which are the smallest number of teeth. I have seen more than one person embarrassed, or even led into the commission of error, by this circumstance."

M. Fardel notices as a condition which he believes undescribed, "a notable increase of secretion from the follicles of the mouth and eye," and which occurs frequently in acute softening and congestion, less commonly in hemorrhage. This morbid state of secretion, he alleges, improves at once with an amelioration of the other symptoms.

*The state of the speech* of subjects affected with acute softening is generally well known; a peculiar state, in which the patient appears not only to have lost the faculty of articulating, but of uttering a sound even, has once at least been noticed by the author. We have also seen this.

When the patient is in a state of complete coma, *the senses* participate in the general abolition of the intellectual faculties. But, under such circumstances, it is not so much abolition of the sense, as loss of consciousness of the impressions transmitted by its organ to the brain, that the observer ascertains the existence of; and, except under these circumstances, the senses do not appear to suffer materially. M. Fardel appears never to have observed deafness, unless where the patient was in a state of coma. Sight is sometimes, but rarely, lost on the hemiplegic side; and it may be perfectly retained in spite of immobility of the pupils.

*The condition of the pupils* is made the subject of lengthened examination, introduced by the observation that in the natural state the pupils are generally very, sometimes excessively, small in advanced age. The statements of Lallemand, Carswell, and the cases of Rostan, Andral, and his own, are analysed, and we cannot exhibit more faithfully the general result than by transcribing the account of the state of the pupils in seventeen of the author's own cases.

| | | |
|---|---|---|
| Pupils natural in | 3 | cases. |
| contracted | 1 | ... |
| narrow | 1 | ... |
| moderately contracted, or rather narrow | 3 | ... |
| very much dilated | 1 | ... |
| dilated, or moderately dilated | 5 | ... |
| on paralysed side dilated ; contracted on the non-paralysed side | 1 | ... |
| dilated on the non-paralysed side ; the cornea being opaque on the other | 2 | ... |

They were besides noted as immoveable in six cases. The pupils are not then habitually contracted in acute softening, as Lallemand and Carswell teach; nor does this contraction or dilatation (as it appears from the facts referred to) present any constant relation to resolution or contraction of the limbs. It is important to observe, that the observations were made at the commencement of the disease, precisely at the period when contraction should, according to the above pathologists, be most obvious. Lallemand has also stated, that the pupils are generally dilated in cerebral hemorrhage. Thirty-one cases (Rochoux ten, the author twenty-one,) give the following results upon this point:

| | | |
|---|---|---|
| Pupils contracted, or narrow in | 18 | cases. |
| dilated | 9 | ... |
| natural | 4 | ... |
| moderately dilated | 3 | ... |

Hence it would appear, that if any positive diagnostic inference were to be drawn from accurate experience on the point, it must be that their contraction would announce hemorrhage rather than softening; but we perfectly agree with the author in the sageness of his conclusion, that the state of the pupils supplies no means of distinguishing the two affections.

*The pulse,* unless in the case of complication, deviates, in the majority of instances, in no particular way from the natural condition. In four only of M. Fardel's cases did fever exist, and in one of these there was pneumonia in the third stage; in the others the state of the gastro-intestinal mucous surface appears not to have been ascertained. Perhaps the latter may be a hypercritical objection in the eyes of some, as there do not seem to have been any notable abdominal symptoms; but cerebral affections so blunt the sensibility in respect of these, that it is not without its importance.

In the brief sections on respiration and nutrition, we discover nothing very remarkable.

*The duration of acute softening*, terminating by death, is calculated as follows from fifty-nine cases, of which twenty-seven are the author's own, sixteen M. Rostan's, and the same number M. Andral's. Death occurred

| | |
|---|---|
| Within the first forty-eight hours in . . | 11 cases. |
| Before the fifth day . . . . | 26 ... |
| Before the ninth day . . . . | 43 ... |
| From the ninth to the twentieth day . . | 7 ... |
| From the twentieth to the thirtieth day . . | 9 ... |

In a brief section the author gives a condensed view of the progress of acute softening, when following each of five types in respect of its symptoms. These descriptions are most graphically drawn up, and we cannot refrain from extracting one as a specimen of all,—it is of the apoplectic form of the disease:

"...... patients suddenly losing the use of speech, of motion, of intelligence, either after vague suffering of some kind, or without any previous indication of the coming malady whatsoever; extended on the back, the face marked by an expression of hebetude, the features distorted, the eyelids closed or scarcely open; they utter not an articulate sound, and occasionally plaintive murmurs alone give evidence that they are not entirely dead to the external world. On one side of the body their limbs, paralysed and inactive, lie extended and flaccid beside them, or flexed and uselessly contracted, almost always deprived of sensibility; the limbs of the opposite side, on the contrary, are moved either from simple restlessness, or in consequence of pain being excited by medical examination, or to perform some act which their remnant of volition permits them to attempt. After some hours, or a few days, a slight remission of these symptoms is generally observed; the eyes open, a certain return to life, if not to sensation, displays itself in the look; a few ill-articulated words are heard; the paralysed limbs recover some little power of movement and sensation. But ere long the respiration becomes embarrassed; the region of the sacrum, suffering under constant pressure, irritated by the contact of the urine and feces, reddens, excoriates, and ulcerates; the powers of motion and sensation are lost again, and now in every part of the body; the faculties of the intelligence and the senses disappear to return no more, and general death soon follows that of the functions of relation." (p. 156.)

Over the other analogous sketches, as well as a valuable section entitled, "Appreciation of the symptoms of acute softening," we are obliged from limitation of space to pass.

The chapter on *the diagnosis* of the malady contains much, which calls for full notice.

(1.) The most characteristic form of softening is that in which the disease commences by gradual alteration of movement and intelligence, attended with perversion of sensation; as cephalalgia, numbness of the limbs, &c. especially when the unnatural sensations in the latter are limited to one side. But if it be true that softening always commences by congestion, we should be inclined, à priori, to affirm that there can be no symptomatic mark of softening at its outset, which shall distinguish it from mere congestion. And such is, in truth, the fact. It is generally the continuance, and especially the persistent severity of the symptoms that announces the addition of inflammation to congestion. The predominance of the symptoms on one side of the body has been generally set down as distinctive of softening, but M. Fardel shows that this circumstance is of little value when the disease sets in suddenly by an apoplectic attack; he considers it extremely rare, however, if the symptoms have affected both sides of the body and been slowly evolved, to discover anything but congestion of the brain or serious meningeal effusion.

As regards cerebral hemorrhage nothing is more rare than a gradual and progressive development or " march" of its symptoms. The whole hemorrhage is not always accomplished at once, but its increase then takes place by *sudden and abrupt attacks.* In this statement of the author we perfectly coincide, and consider it right to point out with him what we believe to be a very serious error of Dr. Copland, (Dict. of Med. vol. i. p. 82,) namely, the assertion that in the " gradually increasing or ingravescent apoplexy" of his classification, " extensive extravasation of blood is always met with."

Hemorrhage into the arachnoid, unfortunately for facility of diagnosis, sometimes follows a course perfectly similar to that of the present form of softening. M. Andral's fourth case, (Cl. Méd. t. v.) furnishes an excellent example of the impossibility of distinguishing such cases of meningeal hemorrhage from softening: luckily this species of hemorrhage, a rare morbid state in the first place, does not often follow the course now referred to; of twenty-four cases only five, according to an analysis by M. Fardel, presented any similarity to the progress of this form of softening.

(2.) We have next to consider the apoplectiform variety of the disease. An apoplectiform attack may be the consequence of various morbid states of the brain, besides softening; of congestion (ictus sanguinis),—of meningeal hemorrhage,—of non-inflammatory sanguineous infiltration of the brain; of serous effusion; of purulent meningitis; and, above all, of cerebral hemorrhage. The author, however, endeavours to establish the distinctive marks of softening and of cerebral hemorrhage, and we shall confine ourselves to an abstract of his observations upon this head. The apoplectiform manner of attack in softening is by M. Fardel referred to the congestion (intensely developed) forming its first stage;—a perfectly fair explanation, as " apoplexy" is well known to be the occasional evidence of mere congestion. In these cases then the mode of attack is identical; is their subsequent course different? Cruveilhier, (Anat. Path. livrais xxxiii.) believes that in some cases the diagnosis is only to be made in the dead-house. We shall one by one pass in review certain circumstances said to be more or less distinctive.

*a.* It has been said that in hemorrhage the symptoms acquired from the first their maximum intensity, whereas in softening they commonly increase by degrees. The statement is true in a certain number of cases; but its fallacy, if made of general application, is well exhibited by M. Fardel. " When," he remarks, " general congestion supervenes abruptly, and partial softening takes place, while the patient is still under the influence of the congestion, death occurs in some cases before this latter has disappeared, and the proper symptoms of softening must then have been masked by it; in other cases death is slower in occurring, and to the general symptoms of congestion will succeed the partial symptoms of softening,—now under these latter circumstances especially, the successive diminution of the symptoms perfectly simulates the course of hemorrhage." (p. 187.)

*b.* The existence of precursory phenomena has been considered of great value as a guide to the diagnosis of softening; but as commonly understood, we have already seen from M. Fardel's researches, these phenomena are nothing more than symptoms of an actually existing softening of the gradually developed form, quite a different one from that at present under consideration. He himself is inclined to believe, but he advances this with reserve, that true premonitory symptoms are more common in cerebral hemorrhage than in apoplectiform softening.

If convulsive movements be observed after an apoplectiform attack, the case may be set down as one of softening; however, in order to make this proposition strictly correct in respect of *primary* softening, it is necessary to add that the convulsive movements occur at the outset, if at a later period they might depend upon a *secondary* softening developed around coagula.

It has been said that if the senses be preserved at the moment of attack, the case cannot be one of congestion or hemorrhage; some rare facts show that even this proposition is not universally true, but unquesionably if the intellect be unaffected in an apoplectiform attack, the presumption in favour of its depending on a softening is exceedingly strong.

If an individual labouring under an apoplectiform attack, give evidence of spontaneous pain, or of unnatural sensibility of the skin in the paralysed limbs, the case is certainly one of softening. But these phenomena are very rarely observed in the present form, and belong to the "gradually increasing" and "ataxic" varieties. Retained sensation simply is by no means a distinctive sign of softening; in fifty cases of the disease collected by the author, MM. Rostan and Andral, the cutaneous sensibility was only retained in twenty, in the remainder either entirely impaired or altogether abolished; in twenty-one cases of cerebral hemorrhage by the author, it was retained or but very slightly impaired ten times, completely paralysed in the remaining eleven.

Persistent contraction (*contracture*) has, as is well known, been set down as almost pathognomonic of softening, and its connexion with cerebral hemorrhage scarcely investigated at all. We long since observed cases exposing the complete fallacy of the exclusive doctrine referred to, and the observations of M. Fardel perfectly coincide in nature with, and have much more numerical precision than our own. Now of forty-seven cases of acute softening attended with paralysis, (Rostan, Andral, the author,) *contracture* attended only thirteen; whereas the author finds this symptom noted in nineteen of twenty-nine cases of cerebral hemorrhage observed by himself. But this latter position requires qualification. "Whenever," says M. Boudet, in his treatise on Meningeal Hemorrhage, "the cerebral substance alone is implicated in hemorrhage, so long as no inflammation arises around the effused blood, no *contracture* occurs. But whenever, in addition to lesion of the cerebral substance, rupture of the walls of the ventricles takes place and consequent effusion of blood into those cavities, or on the surface of the brain, *contracture* follows."

This very interesting statement M. Fardel regards as generally true, and upon it may, in many cases, be satisfactorily established the diagnosis of secondary ventricular hemorrhage. Now some propositions respecting the general question before us may be deduced from what has just been said.

When slight apoplectic symptoms coexist with *contracture*, the diagnosis must be softening; because, the fact of *contracture* existing excludes the idea of hemorrhage into the substance of the brain alone; and secondly, the slightness of the symptoms is incompatible with the idea of ventricular hemorrhage from rupture. Again, when symptoms of much severity, announcing a considerable amount of compression, are not accompanied with *contracture*, we are justified in affirming that we have a case of softening before us: because the absence of *contracture* excludes the idea of ventricular hemorrhage; and the severity of the symptoms seems incompatible with the mere existence of effusion into the proper cerebral substance. These marks of distinction are, as M. Fardel admits, minute and perhaps in not a few instances destined to prove far from satisfactory, nevertheless they appear the only ones that can be established; and, as he very justly observes, it is not his fault that the line of distinction between the two maladies is not more clearly defined.

(3.) When acute softening follows the "ataxic" course of M. Fardel, that is, when phenomena of paralysis and excitation, &c. are combined, there is no chance of confounding the case with one of cerebral hemorrhage, but the similarity to meningitis is most puzzlingly close.

In a certain number of cases of softening delirium is the only cerebral symptom; the same is true of meningitis; are there any means of predicating with certainty the source of the delirium in the two instances? None of a positively distinctive character. Nevertheless it is true that the delirium of meningitis is more violent, more noisy than that of softening; the febrile symptoms are more intense in the former than in the latter; and other head symptoms, cephalalgia, photophobia, &c., more prone to coexist, and more severe when present. The age of the subject under observation will sometimes be of material service in guiding our judgment; it is certain that acute meningeal affections are singularly rare in subjects of advanced age, and that in children the constant tendency of encephalic inflammations is to occupy the membranes.

Convulsions, *contracture*, muscular trembling, pains in the limbs, and impaired motility occur too frequently in meningitis to be of great utility in fixing the diagnosis on softening; but if limited to one side of the body they are useful, because in meningitis they almost invariably affect both. In the exceptional cases of meningitis in which it has been found that such symptoms were limited to one side, (Andral, Cl. Méd. t. v. p. 42,) there has always been, we venture to affirm, as in the case now referred to, local cerebral congestion, that is an early stage of softening, to account for it.

M. Fardel's general conclusion is that when either of the two morbid states exists alone, "the general aspect of the patient and the habit on the part of the physician of seeing such cases will be more useful in supplying him with a diagnosis, than all the rules that could be traced beforehand."

Chronic softening. We have now brought to a conclusion our analysis of the first part of the author's volume, and completed the subject of acute softening; the second part introduces us to the chronic form of the malady.

Chronic softening exhibits itself under certain anatomical forms, which the author refers to successive periods of development: the first period (a), pulpy softening; the second period (b), marked by different conditions according as the convolutions or deep-seated parts are implicated; a third period (c), distinguished by the disappearance of the softened pulp. We proceed with the author to consider each of these periods in detail.

a. When softening passes into the *chronic* state, it consists at first like the acute disease, in a simple diminution of consistence, but is distinguished from it by the absence of redness. Those acquainted with the general state of opinion on this subject will at once recognize the fundamental difference of this mode of viewing the nature of white or colourless softening of the brain from those of preceding writers. We simply draw the reader's attention to this point for the present; the progress of our notice will fully develop the subject.

The appearances in white softening were, as is well known, pertinaciously attributed by Lallemand to infiltration of the cerebral substance with pus. It is sufficiently remarkable that a dead white colour and a bright orange yellow, (which we have seen really depends upon infiltration of blood,) should have been both referred to the same cause, purulent infiltration; but this is not theonlypoint in his doctrines which indicates facility in theory, rather than soundness of logic on the part of the Montpellier Professor. Softening or inflammation of the brain we believe M. Fardel to be perfectly correct in affirming is most rarely attended with suppuration, unless when the effect of injuries or disease of the cranial bones. Simple infiltration with pus does not occur in chronic encephalitis; if pus exist under these circumstances, it is collected into abscesses.

Microscopical examination was, as the readers of this Journal are aware, applied some while past by Gluge to the substance of softened brain. We analysed his paper, (Brit. and For. Med. Rev., vol. X, p. 259, July, 1840,) rather because it recorded the results of the first attempt of the kind, than in compliment to the fulness of the investigations or soundness of the conclusions reported. Both are examined by M. Fardel, and the complete want of harmony between Gluge's first conclusion, "pus is present in many cases of white softening," and his facts made (ex ore suo) obvious. Not contented with this, M. Fardel and "his friend Dr. A. Becquerel," must examine for themselves the micrology of the matter. And a precious business the two friends made of it; but not a whit greater is the absurdity of their descriptions than might be anticipated from two babes in the wood like themselves, with just as much knowledge of the microscopical anatomy of the brain, as of last month's fashions among the belles of the moon. There is assuredly something *sui generis* in the unblushing vanity of a Frenchman; fancy, if you can, a young man belonging to any other nation in the world but the *grande* one, taking up a microscope, perhaps for the first time in his life; for aught we know, or he proves, turning it wrong side uppermost as he used it; applying it to the investigation of a most complicated morbid state of the tissue, the most difficult, even in the natural state, to unravel in the whole body; printing a page of his comically absurd "results," and then coolly telling you at the end of it, "I dare not place *too much* confidence in results so different from those obtained *up to the present time* (!) by different micrographers,"—Valentin, Müller, and such small people alluded to by name. By all our—but we must not disgrace the pages of the "British and Foreign" by swearing; we swallow our wrath and pass on.

The redness of acute softening is produced by congestion or infiltration of blood; in the latter case the chronic disease will exhibit a yellow colour, deep in proportion to the quantity of blood infiltrated: in the former the congestion must disappear after a certain time, and the softened tissue recover its natural hue. And not only will the injection diminish, but the very vascularization of the part; the vessels appear in some cases to disappear altogether. Hence parts in a state of chronic softening never—setting aside some infinitely rare examples—present a red colour or become the seat of hemorrhage. In some instances softened parts in which the disease is of old date, present an interlacement of long and good sized vessels, but this is a different thing from a true capillary plexus. According to M. Fardel then, white softening is merely colourless softening, or softening of the part without any unnatural colour being superadded; chronic softening in the gray substance may consequently be of gray, but never of white colour. The same notion was taught by M. Dechambre. In the latter situation, from the frequency of infiltration of blood, it is generally yellow.

Pulpy chronic softening occurs in all parts of the brain: but is less commonly observed on the surface of the convolutions than elsewhere, on account of the rapidity, according to the author, with which the disease in this situation tends to pass to a more advanced stage.

General softening of the brain, a condition which when not preceded by cerebral symptoms we believe to be of cadaveric origin receives some consideration at the hands of M. Fardel; but no new light is thrown by him on the subject.

B. Portions of brain in a state of chronic softening may remain for almost an indefinite period in the pulpy condition just described; but this is rare. At least, according to M. Fardel, when the duration of softening is prolonged, the softened part undergoes certain transformations. These transformations are of a completely different nature in the cortical substance of the convolutions and in the deep-seated parts.

In the cortical substance the tendency is to the production of yellow patches extending over two or more convolutions; flattened; finely tuberculated; remarkably coherent, sometimes so hard as to be with some difficulty torn; on the surface appear very minute vessels, adherent to the pia mater or not. Sometimes *softening*, "less advanced," (a curious contradiction in words, for as every one knows the yellow patch is in a state of induration,) surrounds them; in other cases there is actual destruction of substance in the subjacent cortical substance, encroaching, it may be, on the white. These ulcerations are, according to M. Fardel, the last stage of chronic softening: they are rarely observed; the alleged reason is either that a suspension of the morbid process or the occurrence of death interferes with their production; this is merely stating the fact in other words. The subjacent medullary substance is rarely healthy under the yellow patches; it is generally in the state to be presently considered. The description here given of the yellow patch of the convolutions contains nothing particularly novel, although the author seems persuaded that it possesses this merit in the very highest degree, while at the same time he admits the morbid appearance has been accurately figured by Cruveilhier, and described by a M. Bravais. Dr. Carswell too has figured the same condition.

We have next to consider the changes in softened medullary substance. The medullary substance disappears over a variable extent of surface, and its place is found filled by loose cellular tissue, exhibiting irregular meshes filled by a turbid and whitish liquid, mixed or not with flocculi which appear to be nothing more than fragments of cerebral pulp.

Of the frequency of this condition, to which he gives the name of "cellular infiltration," the author is persuaded, he himself possessing more than forty cases of the kind, and believing that in some additional instances it must have escaped him. The general seat of this state is the white substance of the hemispheres; it frequently occurs also in the corpora striata, but the author has never met with it in the optic thalami; nor has he seen it in the cortical substance of the convolutions, the secondary changes in which locality have already been shown to be of a different description. It occurs in the cerebellum, also in the medulla spinalis; and is, to say the least, like softening itself, extremely rare in the medulla oblongata. The extent of "cellular infiltration" varies from that of a nut to a mass the size of one or two lobes.

The cellular tissue of the part appears to M. Fardel to be the natural cellular constituent of the brain, exposed by the destruction of the nervous substance. It exhibits itself in the form of filaments, threads, bridles, crossing the space in which it exists, in all directions. It is generally of white colour, though sometimes a little grayish; sometimes it exhibits a slightly yellowish tint. Vessels generally of large size are seen in it in the greater number of instances; as before explained a capillary plexus is not discernible.

In consistence this tissue varies. It may be extremely delicate and friable, which indicates in the author's estimation that it is itself about to disappear as a further phasis of evolution of the disease: or in other cases it is so hard as to offer the resistance almost of fibrous tissue; which state the writer regards as an evidence of a species of cure, "the sole one possible in the case of lesions so advanced." The size of the meshes, depending as it does upon the amount of absorption both of the nervous substance and of the cellular tissue, supplies a measure of the advancement of the disease; the larger they are, the further has the morbid state advanced.

The fluid filling the meshes (whey-like fluid of Andral, milk of lime of Cruveilhier,) resembles very closely in general appearances the liquid with which it is compared by Cruveilhier. We consider it unnecessary to rehearse the author's reasons for not considering it purulent; as its characters are in every respect opposed to those of matter of that description. The author regards it as the produce of a complete liquefaction of cerebral substance; a notion, he says, confirmed by the results of experiment,—he has found that cerebral matter triturated with water yields a fluid precisely resembling that now under consideration. He appears to consider that there is no serosity thrown out into the softened part, but that the whole of the fluid arises from simple liquefaction of the brain : this we do not believe ; that it should be so, is as incomprehensible as opposed to the general law, well known to pathological anatomists, affecting the progress of things in the brain, whenever a tendency to empty space in the cranium is produced by changes in that viscus.

The author indulges in a very characteristic fling against pathologists in general, for their remarkable ignorance of the condition which he has baptised with the name of " cellular infiltration." Upon this we beg to offer a few words. It is utterly and wholly irreconcileable with fact that the total description given by the author was not known before he wrote. There is not a syllable in the mere anatomical facts he relates, which we did not know as well before we saw his volume, as now after its perusal, and *we* learned what we know from writers all of them occasionally (especially when any of their doctrines can be twisted into food for censure,) quoted by M. Fardel, from Lallemand, Foville, Andral, Cruveilhier, Carswell, and above all the late Dr. Sims, whose admirable papers in the Medico-Chirurgical Transactions contain all that we have yet seen of *positive* information upon this particular state of the brain after softening. As regards the catenation of the different morbid states taught by M. Fardel, and which may be novel somewhat, that is another point. Then in respect of the name he choses to give the condition, we venture to affirm he could with difficulty find two words less capable of accurately expressing, or less suggestive of the state than " cellular infiltration."

We pass over some intermediate matter and take up the pages of our author again, where we find him engaged in the discussion of a very interesting question in morbid anatomy, the distinction between hemorrhagic cavities and those produced by softening. His tendency to self-glorification leads him at the very outset of the inquiry into an obvious mistatement, " the morbid changes I have considered [cellular infiltration and chamois-leather patches] have been *up to the present time* generally considered as traces of former hemorrhages." Now if he had said they were *formerly* so considered, he would have been right; but there is not a single person whose general acquaintance with morbid anatomy is sufficient to place him in the class of well-informed physicians, that does not know sufficient of the researches of the writers named a few lines back, to be alive to the fact that the changes in question are not necessarily the result of hemorrhage, and are sometimes at least that of softening. This is putting the matter in the least dogmatic way; but it may be inquired with M. Fardel, whether the changes in question can at all, under circumstances of a certain kind, be ascribed to hemorrhage ? To examine this question requires a preliminary study of the successive periods of cerebral hemorrhage. In this M. Fardel engages; and we shall follow his example. First, however, the author informs his readers to whom is due the gradual establishment of the notion that all cavities found in the brain are not the result of former hemorrhages: the examination is just what we should have expected, the name of Sims carefully omitted, although elsewhere as carefully quoted, where there seemed a possibility to the honest author to connect its mention with error on the part of its owner. But we forget Sims was not a

Frenchman, and upon earth what matters it, then, what he said or did?

One form of hemorrhagic cavity, according to general descriptions, is that in which the cavity is traversed by filamentous bridles in various directions. The existence of these bridles appears to M. Fardel to exclude the idea of hemorrhage, for the following reasons: If blood be absorbed so perfectly as to leave no trace of its presence, it must generally leave a cavity, accurately circumscribed, perfectly free from cerebral substance. If the walls of this cavity approximate, however, they may become united by means of adhesions; but in this case the said adhesions will retain the walls in contact with each other, and instead of a cavity a cicatrix will form, or at the most, a few isolated bridles may stretch from one wall to the other, but this is a very different thing from the abundant supply of cellular tissue in the cavities in question. The notion that the adhesions are the result of organization of the clot itself, the author rejects as altogether unproved, and affirms that the real course of things in the case of effused blood is, as far as observation goes, altogether different. To this latter point we shall presently return; meanwhile we may state that M. Fardel with much justness objects in toto to the manner in which the conclusion has been drawn, that all cavities in the brain are the consequence of previous hemorrhage. The true method of ascertaining the alleged fact would have been to have observed a certain series of recent hemorrhages proving fatal at different periods, and on the condition of the parts in these cases to have founded the evidence for the statements made respecting the transformation of effused blood, and the generation of cavities divided into meshes by a network of new formation. This, according to M. Fardel, has never been done. The sole observations made, he says, have been instituted on the bodies of persons dying a long period after an attack of apoplexy or something of the kind,—for in the greater number of cases the previous history of the individual is exceedingly vague. Now every one knows the frequency with which an apoplectic attack may be the result of acute softening. There is one natural difficulty connected with the subject, and this is, that hemorrhage rarely destroys life, except at the first or at a very distant time from the attack: it rarely proves fatal (and then generally from fortuitous causes) during the intermediate time. With some difficulty, M. Fardel informs us, he has collected a series of cases, with the date of seizure, of death, and the condition of the blood carefully ascertained. The duration of the disease had in these cases, seventeen in number, varied between thirty days and several years: the increase being gradual from the first case of thirty days' duration to the sixteenth of twenty-one months. Now we cannot extract the details; but we are bound to say that the conclusions drawn by M. Fardel from them are justified by the facts. They show it is impossible to affirm at what time effused blood shall cease to present the characters of that fluid; that this time may be influenced by a variety of circumstances, such as the extent of effusion, the nature of the blood, the general constitutional state, the regimen and treatment, possibly too, to which the patient may have been subjected. And it appears certain from the cases collected, that the physical characters of blood may be recognized in effused fluid several months, a year even, after its extravasation. Hence, then, it would appear altogether unjustifiable to set down simple cavities containing no blood found in the brain a month, six weeks, or two months, &c., after an attack of apoplexy, as proving that that apoplexy was hemorrhagic. In the case of undoubted hemorrhage, sometimes the solid part of the blood separates from the liquid, so that a hard central mass is found separated by serosity from the walls of the cavity containing it; in other cases, on the contrary, the blood is uniformly converted into a thick muddy matter which liquefies and gradually undergoes absorption, without appearing, according to M. Fardel, even susceptible of organization. We regret that we are unable to condense the very close and judicious criticism to which several cases of alleged hemorrhagic cavities published by M. Rochoux are submitted.

c. The third period of chronic softening is represented by the disappearance of the softened substance. In the yellow patches of the convolutions, these parts shrivel or undergo complete alteration of shape; the cortical layer becomes thin, the yellow patches themselves often eventually disappear, and in many cases all that can be detected is a yellowish discoloration of the surface of the medullary substance. In the softened medullary substance the further progress of things beyond the state of "cellular infiltration" last described, is marked by the gradual increase of size of the meshes, and eventually the disappearance of the cellular tissue forming their walls.

Now in the case of the yellow patches of the convolutions, the appearances produced in their ultimate stage now referred to have been known as those of ulceration of the brain. In five cases of the kind related by the author, these ulcerations varied in diameter from that of a shilling to that of a five shilling piece; were of irregularly round form, having well-defined borders cut perpendicularly to the surface, and equalling in depth that of the cortical substance itself. They were generally covered at the fundus by a membrane which was always smooth, sometimes fine and transparent, or thickish and vascular, sometimes of yellowish hue. The pia mater adhered to them but slightly, and neither it nor the arachnoid presented any particular change in the parts corresponding to them. The medullary substance underneath was either healthy or in a state of pulpy softening or "cellular infiltration." The author signifies in a note that the term ulceration of the brain has generally been applied to an alleged acute morbid change, which he has himself never seen. Nor have we. But we would observe that the chronic condition to which he gives the name of ulceration, as above, was excellently well described by Dr. Sims, and its relationship to softening of the convolutions successfully traced: if the *name* be new, the *thing* then is not so, which strikes us as being rather the more important of the two. Nay, further, we altogether doubt the correctness of the term ulceration as applied to the state described; that the removal of the patch is effected by a process analogous to that of common ulceration is anything but proved, and to say the least extremely doubtful.

The conditions of the medullary substance described by M. Fardel as resulting from the progress of softening after the stage of "cellular infiltration," are, we readily grant him, in all probability, really referrible thereto, and we perfectly acknowledge the justness of his censure of those writers who have indistinctly applied the term atrophy to these and various other more or less dissimilar states. And it must be confessed that morbid states so extensive and complete as are exemplified in the following case could only be traced to their primary cause by a series of observations of the greatest minuteness and fullness of detail: the mere consideration of the single case could never clear up its difficulties. The case we refer to is given by Andral (Clin. Méd. xv. p. 618). A man died at the age of twenty-eight, of peritonitis; at ætat. three he had fallen on his head from the first floor into the street, and became in consequence paralysed on the left side. Some changes, unnecessary to enumerate, occurred in the state of movement subsequently with the progress of time. But he had never suffered from intellectual deficiency in consequence of his fall; his speech was easy, his intelligence that of ordinary men. The meninges on the right side were transparent and fluctuating; when incised, a great quantity of limpid water-like serosity spouted forth; between the membranes and the lateral ventricle no trace of nervous substance was to be detected, a vast cavity occupied the space between them. This condition M. Andral calls atrophy of the brain, but believes that it was secondary to inflammatory changes supervening on the original fall; M. Fardel regards it as the ultimate condition of inflammatory softening, as the last link in the chain of morbid changes produced by encephalitis, as an absorption, not an atrophy. The previous links in the chain have been successively described.

The seat of chronic softening is next considered and compared with that of acute. In 181 cases of softening, the convolutions and central deep parts were affected in the subjoined proposition; one hundred of these cases were observed by the author, the remainder are borrowed:

| | *Convolutions.* | *Corp. Striata, and Opt. Thalami.* |
|---|---|---|
| Acute softening | 59 | 30 |
| Chronic softening | 60 | 28 |
| | 119 | 58 |

The almost identity of the numbers of the cases of acute and chronic changes in each of the above divisions of the brain, establishes in the author's belief a strong presumption that the connexion he teaches between acute and chronic softening is real. Again, the number of instances of softening of the corpora striata and optic thalami, appears precisely half that of those occurring in the convolutions, both in the chronic and acute disease; whereas, as is perfectly well known, hemorrhage is vastly more common in the corpora striata and optic thalami than elsewhere. Now almost all the changes attributed formerly to hemorrhage were seated towards the periphery of the brain, an obvious reason for referring them, as the author has done, (and others, as we have seen, before him,) not to that morbid state but to softening. In ninety-five cases of chronic softening:

| | |
|---|---|
| The posterior lobe was affected | 18 times. |
| middle | 51 ... |
| anterior | 13 ... |
| posterior and middle | 7 ... |
| posterior and anterior | 2 ... |
| middle and anterior | 2 ... |
| whole convexity of a hemisphere | 1 ... |
| middle line | 1 ... |
| | 95 |

Softening of the corpora striata and optic thalami are included in those of the middle lobe.

As regards the frequency of the two different kinds of lesion in one or other or both hemispheres, there appears to be no great difference observable.

| | *Softening.* | | *Hemorrhage.* |
|---|---|---|---|
| Left hemisphere | 69 | ... | 43 |
| Right ditto | 71 | ... | 40 |
| Both ditto | 26 | ... | 6 |
| Middle line | 3 | ... | 0 |
| | 169 | | 89 |

We consider it the more important to give these numerical statements, as it has been said by Portal, Gendrin, and Morgagni, that hemorrhage was much more common on the right than on the left side.

*Symptoms.* Passing to the study of the symptoms of softening, M. Fardel introduces the subject by some considerations, very judiciously put, respecting the difficulties of diagnosis of diseases of the brain generally. To these we shall not refer farther. But we find as additional introductory matter, a disquisition upon the nature and reality of two orders of cases, bearing so directly upon the question of the diagnosis of softening that we cannot pass them by. The first order of cases includes those wherein distinctly apoplectic and fatal symptoms have existed, and no appreciable organic lesion been found to explain them. The second order embraces cases in which softening has been found in subjects who during their life exhibited no symptoms giving evidence of such disease. In the first class of cases the cerebral symptoms appear to be sometimes sympathetic of visceral inflammation; in old subjects, of pneumonia for instance. It has been thought by many persons that in these cases (the nervous apoplexy of older authors) cerebral congestion has really always existed. M. Fardel believes, and we are persuaded he is right, that in a great number of cases of the kind, this is the true explanation of what is observed. For it must be recollected that congestion of the brain may occur under two forms, that indicative of increased arterial circulation, and that marked by an engorgement of the venous system with excess of serosity within the cranium, and paleness of the brain. But if it be true that a certain amount of serosity indicates the preexistence of congestion, how is this amount to be determined, when it is well known that the limits of health are extremely variable in this respect? and certainly there are cases in which nothing in the appearance of the cranium or its contents justifies the notion of previous congestion. Is it fair to suppose with M. Gendrin and others, that the signs of that state have disappeared after death? May we say that the brain has absorbed serosity which had been effused, and that congestion has disappeared in the same way as it is known to do in the case of the skin? M. Fardel thinks it next to impossible to admit the post-mortem disappearance of cerebral congestion, because it has been shown by some experimentalists that the phenomenon of removal of redness depends on the direct influence of atmospheric pressure. As regards the absorption of serosity by the cerebral substance, the experiments of M. N. Guillot appear to render it tolerably certain. He finds that the ventricles are filled and even distended with serosity during life; that the quantity of this fluid is found to have decreased in proportion as the examination is made at a

4293

more distant period from death; that this serosity is recoverable in the cerebral substance; that a piece of recent brain will absorb its own weight of water or serosity; that this property varies in intensity with age and other circumstances. Dr. Paterson, in a recent number of a contemporary journal, has described results remarkably similar to these.

Notwithstanding all this, M. Fardel considers it absolutely necessary to admit that a certain modification of cerebral functions may occur perfectly simulating true apoplexy. Now from this fact he would infer that "when after acute apoplectic symptoms, nothing is discovered in the brain, except softening of chronic aspect, it is wisdom to refrain from refuting the symptoms necessarily to the morbid change detected." This would be all admissible, if the notion that the white softening, to which he evidently refers, is a mere chronic form of the red were thoroughly established; until it is, the above statement involves a *petitio principii.* However we shall see, nay, we have seen, that this doctrine is very likely to be the true one.

The author proceeds to consider each particular symptom of the *chronic* malady. In the majority of, but not in all, cases, the power of motility is more or less completely removed from the influence of the will; in some cases simply weakened or abolished; in others modified in various ways, so, for instance, as to exhibit itself in relaxation, *contracture,* trembling, convulsive movements, &c. Relaxation of the limbs is rarely complete in chronic softening; there is almost invariably some remnant of power of motion retained, enabling the patient partially to withdraw the limb when pricked with a pin or otherwise irritated.

*Contracture* is more common than relaxation; sometimes it appears at the outset, and disappears to be succeeded by relaxation; sometimes, on the contrary, it follows the latter, precisely as in cases of softening following hemorrhage. In some instances, again, it is of intermittent existence; in others persistent from first to last. It occurs with greater certainty when the disease has been developed slowly. Under these circumstances it first affects one or two fingers, and progressively gains the hand, wrist, and elbow. In its most highly-marked state, the leg is seen forcibly flexed on the thigh, &c. The stiffness generally disappears some few hours or even days before death. This condition may exist in the non-paralysed limbs; but this is infinitely rare. M. Fardel gives a very useful enumeration of the various states of movement observed in forty-three cases:

| | | |
|---|---|---|
| Movement wholly unaffected in | 10 | cases. |
| Impaired power of motion, without actual paralysis | 6 | ... |
| Spasmodic movements, without paralysis | 1 | ... |
| Simple relaxation, without *contracture* | 11 | ... |
| *Contracture* | 16 | ... |

Hence it would follow that, contrary to what is observed in the acute disease, *contracture* is more common than relaxation in the chronic malady.

*The motions of the tongue* did not appear habitually affected, although articulation often did. This we have observed ourselves also; the difficulty of speech M. Fardel considers produced by forgetfulness of words in the greater number of cases.

*Modifications of sensibility* are among the first conditions which excite the attention, when the disease commences gradually. Numbness, formication, or pricking sensations, sometimes extremely disagreeable, are experienced in one or both limbs of the side to be subsequently paralysed. They are, M. Fardel conceives he has observed, more marked when eventually to be followed by *contracture* than by simple relaxation. Sometimes these morbid feelings occur on both sides of the body, whereby their development appears traced to general congestion.

*Actual pain* is rarely felt in the limbs at the outset; almost always at a rather advanced period, and then associated with *contracture.* Sometimes these pains are excessively severe; they are influenced by change of weather.

Whatever be the amount of *paralysis* or contracture, the sensibility may remain perfectly natural. Under all circumstances, complete anesthesia is extremely rare; and M. Fardel is not aware that anesthesia has ever been observed in chronic softening independently of paralysis of movement.

*Cephalalgia* existed in twenty-four of fifty-three cases observed by the author or MM. Rostan and Andral; it is therefore more frequent now than in the acute stage. It may exist from the commencement, and even, when the disease follows a gradual course, be its first symptom. It may have existed as a premonitory sign and cease when the disease is actually formed. It varies much in intensity and in character; is almost always frontal, sometimes general, rarely limited to one side of the head. Vertigo is in some patients a symptom of very frequent occurrence; varying in amount from a very slight dizziness to complete loss of senses.

Upon the state of the intellectual faculties we do not discover any information requiring us to stop. The intellect may be almost unaffected, but this is very rare.

M. Fardel has, like other observers, met with *peculiar conditions of speech,* which are perfectly inexplicable. A woman invariably added, at the end of every three or four words she spoke, the phrase "*par le commandement;*" and this for several consecutive years. A woman, aged sixty-eight, uttered only incoherent sounds, always the same, and forming together the word *simona* or *chinona;* she heard perfectly, understood what was said to her, but answered everything by this single word, curiously varying the inflection of her voice, however, according to the desire she wished to express. Another patient, whose intelligence is not affected, can say nothing but "*madame été.......... mon Dieu .......... est il possible .......... bon jour, madame.*" These words she pronounces with perfect precision.

M. Fardel refers all cases of chronic softening to the following four forms: 1st. Softening displays itself from the first as an essentially chronic disease. Attended with a variable number of symptoms, it advances slowly or by abrupt increases, but always progressively. 2d. Softening commences suddenly, like cerebral hemorrhage, of which affection it simulates the subsequent progress with great exactness. 3d. The disease commences without determining prominent or, at least, characteristic symptoms; then suddenly gives rise to derangements of function, which run a very rapid course, and quickly put an end to existence. 4th. Softening determines no appreciable symptom; death is the result of some malady or circumstance altogether unconnected with the cerebral affection. (Latent softening.)

Nothing can be more variable than the *duration* of chronic softening. No limit can be fixed between one or two months and a great number of years. Many so-called atrophies of the brain (of which we have noted an instance) met with in individuals of adult or advanced age, are nothing more than the conditions produced by softening, which had occurred at a very early period of life. The only proposition we find in the author's book, which, in connexion with the question of duration, may aid the observer in forming a prognosis, is that where softening, passing to the chronic state, makes incessant progress to the time of death, and that this appears produced by it directly, the disease generally proves fatal within a year. But, on the other hand, when softening, and this is very common, ceases to exercise any very evident influence on the rest of the organism, and itself makes extremely slow or almost no progress, death is rarely produced directly by it. In reality, too, there is not much useful information conveyed by these statements.

The cases and reflections which here follow, illustrative of the four different forms of the disease above enumerated, want of space obliges us to pass over without further notice; they will amply repay close study.

We reach the chapter on diagnosis. The outset of the disease, being that of the acute form, has already been considered; what the author investigates in the present place is the method of recognizing the malady at an advanced period of its existence, when the observer may often be unable to obtain exact information respecting the outset, or when an error may have been committed at first in determining on its nature, or when no diagnosis may then have been considered warranted by the character of the symptoms.

We have seen the impossibility in certain cases of deciding, during the first days of illness, whether an existing set of symptoms depends upon hemorrhage or upon softening. Is the distinction always easier at a more advanced period? The essential characters of the progress of hemorrhage tending to recovery appear to be a gradual decrease of intensity of symptoms, until the patient's state becomes stationary, the affected faculties having as completely recovered their integrity as we can suppose compatible with the existence of a lesion cured, but not removed. But unfortunately this mode of progress may exist accurately to the very letter in cases of softening. One of the author's cases in particular proves this beyond question. However, taking a number of cases together fit for the purpose under consideration, the author finds that in the event of cure of cerebral hemorrhage, the following three varieties only are observed: 1st. For a certain period before death, there may be no apparent vestige of cerebral disease. 2d. Speech and mobility do not recover their original correctness and power; this is the most common case. 3d. Hemiplegia or loss of speech remain as complete as at the first; this is the rarest case. In almost all these cases the intelligence is restored, or very nearly so. But what is most striking and really characteristic in cases of this kind, is that a certain degree of weakness or impaired power of the faculties originally affected is observed; never are phenomena of a *different* kind, such as cephalalgia, pains in the limbs,

*contracture*, convulsions, &c. noticed; when such phenomena occur at least, it is in general easy to ascertain that they depend upon some other circumstance than the presence of a cured hemorrhage.

The distinction of tumours of the encephalon from chronic softening is often far from easy. The author found that in three out of seventy-one cases of tumour (tuberculous, cancerous, or other) of the cerebrum, cerebellum, or membranes, there were no symptoms; of all the phenomena observed in the others, cephalalgia appears to have been the most constant and one of the most characteristic; it was noted in sixty-one of the sixty-eight. This cephalalgia, too, is remarkable for its intensity, which is frequently extraordinary, and for its seat; it was generally limited to one side of the head, in fact, in two cases only was it stated to be general. Now, as we have seen, in softening, cephalalgia is rarely limited to one side of the skull. In some cases, cephalalgia was the sole symptom, until a few hours before death, when convulsions, hemiplegia, or coma supervened. In six cases, blindness gradually came on; M. Fardel is not acquainted with a single example of loss of sight as an attendant on softening. The other circumstances (which we can but very briefly indicate) apparently more or less distinctive of tumour are the occurrence of paraplegia; the greater frequency of epileptiform convulsions, which were in twenty cases unaccompanied with paralysis; the last point is very important, as it is singularly rare to observe convulsions without paralysis in chronic softening. We may resume then, with the author, as follows. The prolonged existence of violent cephalalgia, limited to one side of the head, attended or not with vomiting, and blindness or disturbed vision, without paralysis, if it does not indicate a tumour with certainty, at least excludes the idea of softening. If epileptiform attacks are superadded, without paralysis in the intervals, the probabilities are still stronger in favour of the existence of tumour, especially if speech and the intelligence be unaffected. M. Rostan attaches great importance, in the case of cancerous tumour of the brain, to the existence of *lancinating* pains in the paralysed limbs, perfectly different from those occurring in softening. M. Fardel more correctly considers that sharp lancinating pain occurs too frequently in cases of simple softening, to justify the view of their signification taken by that writer.

*The curability* of softening is very fully discussed by M. Fardel. That an anatomical state is capable of being produced, as a consequence of preexisting softening, which is in itself wholly innocuous, seems supported by a mass of evidence which it is difficult to resist. The reader will not expect us to state this fully, for to the predecessors of the author, and not to himself, we are indebted for it; but we shall briefly review the chief points of the subject.

Pulpy softening may become perfectly quiescent, giving evidence of its existence by no single symptom; but there is no evidence to prove that it can directly undergo anatomical cure, without having previously passed into the more advanced stage of "cellular infiltration."

When softening has arrived at this latter stage, it appears, according to the testimony of various writers, to be capable of cure in three different ways. (*a.*) The cellular tissue surrounding the infiltrated part and that which traverses it undergo a certain amount of induration; in some points it even becomes fibrous, and almost cartilaginous; the spaces forming the meshes of this tissue, enlarged by commencing absorption and by actual shrivelling of itself, are filled with milk of lime-like fluid or serosity; the surrounding cerebral substance is either healthy, slightly indurated, or slightly softened. (*b.*) The portion of cerebral substance presenting the cellular infiltration is completely absorbed, and a cavity perfectly circumscribed is left behind. The walls of this are white, and more or less indurated, lined or not with a membrane, full of milk of lime-like fluid or serosity, and contained in the interior of the hemispheres or opening outwards. (*c.*) Finally, this cavity, if it be not very large, may contract by a sort of plaiting of its walls, become obliterated, and give rise to a white, stellate, or linear cicatrix.

Dr. Carswell states that he never met with an instance of cicatrization after softening; but he distinctly describes the other species of curative change.

M. Fardel has an interesting chapter on the *mode of death* of patients with softening of the brain. Such is the independence of organic and animal life in subjects of advanced age, such as came under the author's notice, that it is rare to observe a patient fall a victim directly to the progress of the softening itself. Certain complications (developed, however, generally under the influence of the cerebral affection,) prove the immediate cause of death: these are 1, some other cerebral affection; 2, some pulmonary affection; 3, the formation of gangrenous sores of the integuments. Upon each of these causes of death full information will be found in the original work.

*The prognosis* of softening of the brain is indubitably shown by the researches on the curability of the disease undertaken within the last few years—or rather by the results of these researches, to be not so necessarily hopeless as was at one time supposed. Curiously enough, Rostan, the first describer of the malady, stated twenty years ago his more than suspicion that, being an affection of inflammatory nature in many instances, it might occasionally at least undergo the curative changes proper to inflamed parts. He had no very satisfactory facts to carry out his notion with, though one of his cases certainly exemplifies the mode of cure now admitted. But the strange part of the matter in connexion with Rostan is, that within the last year he has affirmed that the disease is necessarily and infallibly fatal, that the idea of cure is a delusion: twenty years' experience and the researches of English and French observers, appear to have had a very different effect from that which might have been anticipated on the creed of M. Rostan.

There is another point of view in which M. Fardel conceives his own inquiries have demonstrated that the danger of softening is less than commonly supposed. He believes, on evidence which we had not space to lay before the reader, that many cases set down as examples of mere cerebral congestion, and terminating fatally are in reality instances of incipient softening arrested in their first period. This *must* be true, as congestion is in truth the first stage of inflammatory softening, but though this view adds a new principle to prognosis in cerebral affections, it does not alter any previously admitted ones, because former observers, in speaking of the prognosis of softening, certainly made no reference to such cases as are thus adduced by M. Fardel.

The author now considers himself prepared to discuss that much vexed question, the *nature* of softening of the brain; and though his own opinions have been foreshadowed by much that has gone before, we consider it for the advantage of our readers to examine seriatim the objections raised to one at least of the doctrines held by others, and the precise facts (condensed) on which he supports his own.

The notion most commonly entertained on the essential nature of white softening is that it consists of a gangrenous condition of the brain. Abercrombie appears to have originated this idea, which has subsequently, as is well-known, received most active support from Dr. Carswell, and from various other writers of less distinction. The author opposes this view as might have been anticipated, especially as the alleged gangrene is regarded by those writers as analogous to the spontaneous gangrene of the extremities of old people. He denies that the conditions of the vessels which in the limbs produces senile gangrene (ossification *with* obstructed circulation from coagulation or otherwise) is met with in cases of softening with anything like sufficient frequency to justify its being regarded as the real cause of that diseased state. On the other hand he has himself seen a case in which the arteries of the base of the brain were ossified to such an extent, as to be converted into solid tubes, and yet here was no softening. The author here takes occasion to inquire into the accuracy of the common belief that ossification of the cerebral arteries is extremely frequent in subjects of advanced age, and the main cause of the hemorrhages occurring in their brains. The following numerical statement is really of extreme interest, for we are not aware that any fairer ground than the merest and crudest theory has led to the universal adoption of this notion respecting the generation of cerebral hemorrhage.

| | *State of the cerebral arteries in* | | | |
|---|---|---|---|---|
| | 32 subjects dying of various diseases: brain healthy. | 30 cases of softening. | 20 cases of cerebral hemorrhage. | Total in 82 *old* subjects. |
| Healthy | 9 | 6 | 3 | 18 |
| Thickened | 21 | 18 | 10 | 49 |
| Ossified | 2 | 6 | 6 | 14 |
| Partly cartilaginous | 0 | 0 | 1 | 1 |
| | 32 | 30 | 20 | 82 |

In six of the fourteen cases of ossification too the change was in a perfectly incipient state. The present question, however, has less to do with ossification than with any condition capable of arresting the local circulation: thickening then, which was very frequent, is just as important a state. Upon this the author observes that the proportion of cases of healthy arteries and diseased arteries, in the two classes of subjects, (those with healthy and with softened brains,) was almost precisely the same in the series laid before the reader, and that hence it would be perfectly illogical to refer the softening to the accidentally associated morbid state of the vessels.

If obstruction to the cerebral circulation were the cause of softening, we should expect to find that conditions of the heart interfering with freedom of circulation through that organ should secondarily lead to softening; and that ligature of the carotid artery on either side might possibly be similarly followed by that cerebral lesion. Dr. Law, of Dublin, has printed some cases tending (but no more,) to show that such may be the fact in cases of mitral contraction; and M. Sedillot recently made known a case of ligature of the carotid, in which a soft non-coherent state of the anterior hemisphere of the side on which the vessel had been tied was observed. This condition of the brain was, we are willing to admit with the author, different from true softening; but we do not admire his passing by with a very unsatisfactory remark the curious case of Mr. Vincent, in which the patient died on the seventh day after ligature of the right carotid, with hemiplegia of the left side, and in whom pale softening of the right hemisphere was discovered after death.

The author observes upon Dr. Carswell's statement, "to distinguish softening from obliteration from softening produced by inflammation, it is only necessary to ascertain the presence of the morbid state of the arteries which we have described;" that it is tantamount to saying, the only distinction between softening from arterial obliteration and inflammatory softening is obliteration of the arteries,—a notion altogether deficient in logical precision.

Various other notions of less importance tendered in various quarters respecting the nature of the malady are weighed in M. Fardel's balance and found wanting: we, too, regard them as of "light weight," and accordingly pass to the author's own doctrine. In its main features this doctrine has developed itself in the preceding pages: softening is an inflammation, having nothing special in its nature; essentially the same in the young and the old, and whether produced by local injuries or spontaneously developed; attended with redness in its acute stage, a redness which disappears in the chronic and leaves the part colourless. The "white softening" of authors is thus purely and simply chronic red softening. Such is the doctrine flowing from the 472 preceding pages of the author's volume; but at the 473d he puts the very pertinent question, "Shall we then definitively refuse to admit the existence of *primitive* white softening?" To this the author replies, first, by showing that many cases which have been considered examples of such a state were not so in reality: the evidence in his favour is satisfactory; as, for instance, in cases where œdema of the brain has been confounded with softening; and we consider it unnecessary to be more precise in our notice of this part of the reply. Secondly, he replies by admitting that a certain number of cases have been reported, and to all appearance correctly reported, in which, after an attack indubitably acute, white softening has been detected. Shall we admit that in these cases the disease had been latent for a greater or less length of time? M. Fardel does not yield to the temptation thus thrown out; but with more freedom from prejudice than was almost to be expected, admits that such cases are yet open to explanation. May we go further and suggest that they disprove his doctrine as a *general* one, and express our own conviction, derived from previous study as well as from a consideration of all the facts this writer has accumulated, that cerebral softening is not always essentially one and the same lesion.

As some of the details through which we have conducted the reader may have proved somewhat arid, we shall reward him for his diligence with a little amusement. A preposterous chemical theory conceived by

M. Couerbe has M. Magendie—and who more naturally?—for its patron. The brain, according to M. Couerbe, is composed of:

| | |
|---|---|
| Pulverulent yellow fat | Stearonocote. |
| Yellow elastic fat | Cephalote. |
| Reddish yellow oil | Eleencephol. |
| White fat matter | Cerebrote. |
| Cholesterine. | |

The eleencephol dissolves tolerably well the other matters in the brain which give it consistence; it is isomeric with cerebrote. Now, M. Couerbe thinks, according to the cautious Magendie, that this isomerism may serve to explain a very important "physiological phenomenon," namely softening of the brain; for cephalote having the same composition may under some morbid influence become metamorphosed into eleencephol, dissolve the other solids of the brain, and so diminish its consistence!

The only point, connected with the questions of etiology mooted by the author, which we consider it necessary particularly to refer to, is the condition of the heart. In every case in which softening existed in subjects under fifty years of age, coming under the author's observation, the heart was healthy. As respects the state of this organ in old subjects here are his results:

In old subjects dying of various diseases, brain healthy; hypertrophy of the heart in one fourth of the cases.

In old subjects dying of cerebral softening; hypertrophy of the heart in one fifth of the cases.

In old subjects dying of cerebral hemorrhage; hypertrophy of the heart in a little more than one third of the cases.

That hypertrophy of the heart has no influence in producing softening appears clearly from these facts, nor would theory lead to the belief that it has, but rather to the view held, as already mentioned, by Dr. Law.

Respecting treatment, M. Fardel's work is poverty itself; the disease is inflammatory,—ergo, antiphlogistics constitute the rational means of opposing its progress. In saying this we have in truth said all the author teaches. But the fault is not his; and we must confess we infinitely prefer this simple statement to the long tirade of salines, and alteratives, and tonics, and stimulants, and deobstruents, and refrigerants, and carminatives, and sedatives, with all the other *atives* which, to the bewilderment of the student and the scorn of the philosopher, would have been set down by many writers we have in our eye, had it pleased Heaven to make *them* the inditers of a work on cerebral softening.

And here we take leave of M. Fardel. Occasionally he has ruffled the smoothness of our critical spirit, but on the whole impressed us with a high opinion of his powers of observation, his judgment, and his conscientiousness. He has done better than this; he has added to the stock of sound medical literature unquestionably the most perfect history yet produced of the important disease he has described.

